# Hydrothermal Carbonization of Green Harvesting Residues (GHRs) from Sugar Cane: Effect of Temperature and Water/GHR Ratio on Mass and Energy Yield

**DOI:** 10.1021/acsomega.4c01875

**Published:** 2024-06-06

**Authors:** Alexander Portilla-Amaguaña, Juan Barraza-Burgos, Juan Guerrero-Perez, Venu Babu Borugadda, Ajay K. Dalai

**Affiliations:** † Facultad de Ingeniería, Ciudad Universitaria Meléndez, Universidad del Valle, Calle 13 # 100-00, Cali 25360, Colombia; ‡ College of Engineering, University of Saskatchewan, 57 Campus Drive, Saskatoon, SK S7N 5A2, Canada

## Abstract

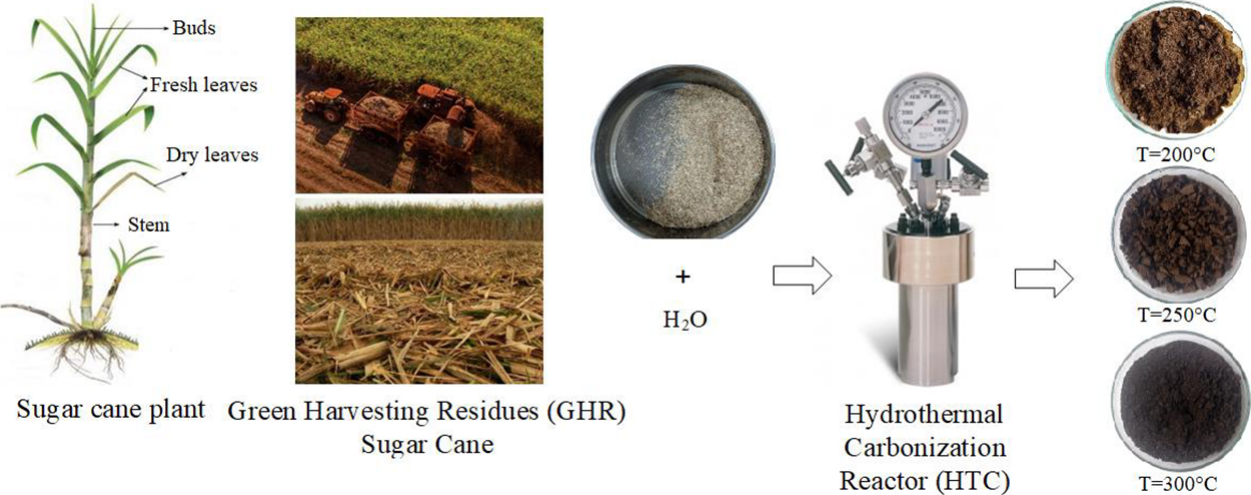

The Valle del Cauca region in Colombia is a significant producer of sugar cane, resulting in large quantities of agricultural residues (green harvesting residues (GHRs)). To ensure sustainable management of these residues, it is crucial to implement proper treatment and disposal technologies while also reusing waste to produce biogas, bioelectricity, or biofuels. The biomass hydrothermal carbonization process offers a means to convert these residues into useful products that serve as fuels or valuable energy materials. This thermal treatment involves the use of water as a solvent and reagent within the biomass’s internal structure. In this study, sugar cane cutting residues were collected with relatively high moisture content of 8.5% wt. These residues were subjected to carbonization temperatures ranging from 200 to 300 °C, along with water/GHR ratios between 5/1 and 10/1. The properties of the resulting hydrocarbons were analyzed by using proximate and ultimate analysis. The objective was to produce hydrochar samples with the highest higher heating value (HHV) and energy density compared with the GHRs. The HHV value of the hydrochar showed a significant increase of 69.6% compared with that of the GHRs, reaching 43.5 MJ/kg. Besides, process parameters were optimized for mass yields, energy yields, and ash content. This exploration led us to investigate a new temperature range between 280 and 320 °C, allowing us to establish an optimal value for the hydrochar’s properties.

## 1 Introduction

Biomass is formed via a chemical reaction between CO_2_, water, and sunlight through photosynthesis, which is the primary process plants use to sustain themselves.^1,2^ During photosynthesis, plants store this chemical energy in the form of biomolecules rich in sugars, such as carbohydrates, while also releasing oxygen into the environment.^3^ The energy is stored within the chemical bonds of adjacent carbon, hydrogen, and oxygen molecules and can be released through digestion, combustion, or decomposition.^4,5^ Lignocellulosic biomass holds significant potential for sustainable energy production and is one of the most abundant renewable energy sources available.^6,7^ The International Renewable Energy Agency predicts a promising future for biomass, projecting that it could account for 60% of global renewable energy end-use by 2030.^8^

Sugar mills are complex industries vital for sugar production from sugar cane, a key ingredient in food and beverage preparation, significantly contributing to the country’s economic development. Sugar cane, a member of the grass family, thrives in many tropical and subtropical regions. It is one of the most efficient plants at utilizing solar energy due to its C4 metabolic pathway, which supports faster growth and higher productivity.^9^

Colombia possesses several regions with substantial potential for agricultural biomass generation.^10^ For instance, annual sugar cane bagasse production is estimated at 1.5 million tons and rice husks at 457,000 tons per year.^11^ Sugar cane residues, particularly in the region of Valle del Cauca and Cauca, which are the country’s two largest sugar cane producers, hold the highest energy potential. These regions have the capacity for 10,000–20,000 Terajoules (TJ)/year in most sugar cane crops and 2000–10,000 TJ/year in others, presenting a remarkable opportunity for the country to produce renewable energy.^12^ The geographic region of the Cauca River Valley, in 2018, processed 95.5% of the country’s total sugar cane across 12 sugar mills, with a total harvestable cultivable area of 207,083 ha, yielding 13.30 tons of sugar per hectare.^13^ The sugar agroindustry generally produces large quantities of agroindustrial waste and by-products at different stages of the production process, including the waste generated during the sugar cane harvest (green harvesting residue (GHR)), which consists of green and dry leaves, and buds left in the field as only the stems are harvested.

**Figure 1:**
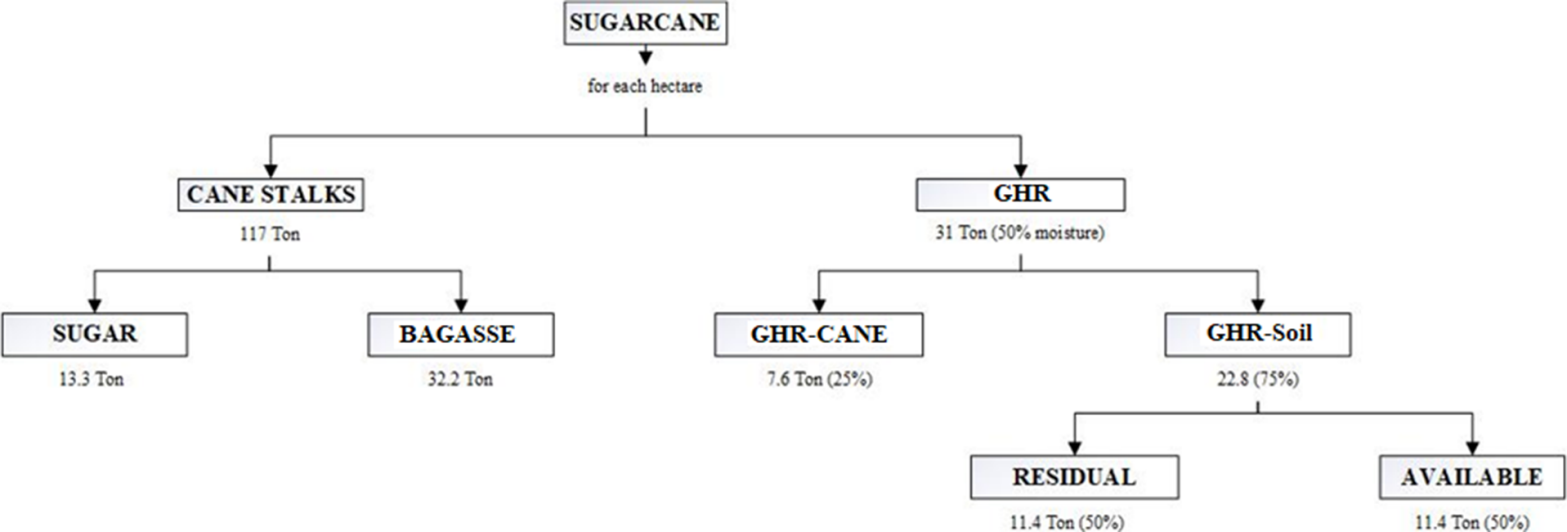
Distribution of renewable biofuel from harvested sugar cane.

Studies carried out by CENICAÑA^14^ report the availability of renewable biofuel for each hectare of harvested sugar cane, from which 117 tons of cane (stalks) are obtained, of which 13.3 tons correspond to sugar production and 32.2 tons to bagasse; from this crop, 31 tons of GHR are also generated with 50% humidity. Of this, 7.6 tons (25%) accompany the cane (stems), while 22.8 tons of GHR (75%) remain in the crop soil, half of which is usable. Figure 1, created based on information reported in the literature, illustrates this behavior more clearly.

The environmental sustainability of the current use of GHRs varies. While some practices, like composting or mulching, contribute to soil health and reduce waste, others, such as open burning, may pose significant environmental concerns such as air pollution. Assessing sustainability requires considering factors such as resource renewal rates, emissions, and ecosystem impacts, which can vary depending on specific management practices and local contexts.

Environmental problems associated with GHRs management include greenhouse gas emissions from decomposition, air pollution from open burning, soil degradation due to improper disposal, and potential water contamination from leachates. Additionally, inadequate infrastructure for collection, processing, and disposal may contribute to environmental and socioeconomic challenges. For example, one of the harvesting methods involves burning the cane prior to cutting; this practice, necessary from an occupational safety perspective, eliminates waste and harmful animals, facilitates manual cutting, and protects the health of cutters as the fluff from the cane affects the skin. However, burning sugar cane causes environmental problems as it deteriorates the soil, pollutes the air, harms the atmosphere, and destroys biodiversity, in addition to its effects on human health.^15^ Hydrothermal carbonization (HTC) offers a solution by converting GHR into hydrochar, a stable, carbon-rich material that can be used as a soil amendment, energy source, or carbon sequestration tool, thus mitigating environmental impacts associated with traditional GHR management practices.

The HTC process aims to replicate natural carbonization by applying heat and pressure to convert raw biomass into carbonaceous biofuel with a higher energy density, resembling coal (hydrocarbonized).^16,17^ This conversion occurs in the presence of water at moderate temperatures (120–350 °C), retention times (5–60 min), and autogenous pressures (2–16 MPa), resulting in reduced O/C and H/C ratios.^18,19^ During the process, various reactions take place, including oxidation, hydrolysis, thermal decomposition, and dehydration.^20,21^ Water plays a crucial role as a reagent, solvent, and catalyst, promoting the hydrolysis and breakdown of the lignocellulosic biomass. One significant advantage of HTC is its ability to convert wet biomass, which typically contains 70% or more water, without the need for drying.^22^ The process involves immersing biomass in water at temperatures between 200 and 300 °C, under saturated pressures of 2–6 MPa, for 5–240 min, in the absence of air.^23,24^ The distribution of HTC products depends on the type of raw materials used and the reaction conditions, such as temperature, residence time, and ash content.^25^

Under HTC conditions, in the presence of water, hydrolysis occurs within the temperature range of 100–374.2 °C and pressures below 22.1 MPa. Water acts as a solvent for insoluble solutes at ambient pressure and temperature, leading to the fragmentation of large biomass molecules and the separation of cellulose from lignin. Lignin serves as a protective layer for cellulose fibers, shielding them from enzymatic, solvent, or other agents’ attacks. Steam separation near the critical point, with or without a catalyst, proves to be an effective pretreatment method, enhancing cellulose reactivity and increasing yields.^26^ Water exhibits the peculiar characteristic of transitioning from a solvent for ionic species under ambient conditions to a solvent for nonionic species under supercritical or near-critical conditions.^27^ Near the critical point, water’s properties, such as the ionic product, density, miscibility, electrolyte solvent power, transport properties (viscosity, diffusion coefficients, and ion mobility), hydrogen bonding, and dielectric constant, undergo rapid variations.^7,28^ The dissociation constant is 3 orders of magnitude higher than at room temperature, making pressurized hot water highly reactive.^28^

The electrochemical properties of water undergo substantial changes, primarily due to increased reactivity in the vicinity of the critical point, with or without a catalyst.^29^ Pavlovič et al.^22^ demonstrated that the ionic product of subcritical water (kw = [H+][OH−]) increases with temperature and surpasses room temperature values by 1–2 orders of magnitude. It reaches a maximum at around 300 °C and then drastically decreases as the temperature rises above the critical point owing to the decrease in ion solvation with decreasing density. These changes in the ionic product have significant implications for acid- and base-catalyzed reactions, which are of great interest in biomass hydrolysis reactions. Similar trends can be observed in mass and heat transfer properties, such as high diffusion coefficients and thermal conductivity (reaching a maximum at the critical state), as well as low viscosity, making water more gas-like than liquid-like.^30^

The mass ratio of water to biomass, also known as the water/biomass mass ratio, plays a significant role in HTC. Subcritical water acts as a nonpolar solvent in hydrothermal carbonization, enhancing the solubility of organic compounds. At high temperatures and pressures, water exhibits both acidic and basic characteristics as it dissociates into hydronium (H_3_O^+^) acid ions and hydroxyl (OH^**–**^) ions. The use of water in the hydrothermal carbonization process is advantageous due to its affordability, nontoxic nature, and natural presence in biomass.^31,32^

This process effectively minimizes the loss of volatile matter and immobilizes organic compounds in the solid products. Moreover, valuable liquid products are obtained, containing dissolved inorganic and organic compounds, such as sugars, furans, furfurans, and organic acids. Aqueous by-products retain inorganic nutrients containing alkali and alkaline earth metals (AAEMs) or their compounds with chlorine and silicon, opening up possibilities for HTC to produce hydrocarbons or form carbonaceous materials in combination with other components (e.g., inorganic nanoparticles of noble metals), thereby creating compounds with unique physicochemical properties^33,34^

Research conducted by Nizamuddin et al.^35^ and Kieseler et al.^36^ indicates that biomass can undergo thermal processing to yield hydrochar, which undergoes structural rearrangement as it degrades into solid, liquid, and gaseous products. The resulting homogenized solid, characterized by a low O/C ratio, serves as a primary byproduct with versatile applications. This suggests that the HTC process is a successful conversion method for upgrading residual biomass and enhancing its energy density.^37^ Temperature emerges as the crucial variable influencing the properties of the produced biofuel.^38^

Energy densification primarily involves the removal of oxygen from lignocellulosic structures through hydrolysis, dehydration, decarboxylation, aromatization, and recondensation reactions. These reactions lead to the formation of a solid product with a significantly higher carbon content compared with the original feedstock.^39^ Ramke et al.^40^ and Parnthong^41^ propose that this technique relies on a straightforward chemical process, where the exothermic dehydration of carbohydrates and the heat emitted by the exothermic reactions within the reactor contribute to maintaining the required temperature during the carbonization process. As a result, the energy consumption for maintaining the reactor temperature is substantially reduced.

The decomposition rate of components in the HTC process is primarily influenced by several key variables: reaction temperature, nature of the feedstock, reaction time, catalyst, pressure, and mass ratio of water to biomass. Temperature holds paramount importance in all hydrothermal processes, especially in HTC. The reaction temperature plays a crucial role in providing the heat required for the disintegration of biomass bonds and the formation of hydrocarbons, which ultimately determines their physicochemical properties.^42−45^ Nizamuddin et al.^35^ indicated that cellulose hydrolyzes significantly in the range of above 200 °C, hemicelluloses around 180 °C, and lignin degrades at approximately 200 °C. The reaction time can range from several minutes to a few days; however, beyond a certain time interval, it has no significant impact on the hydrolysis reactions.^46^ Feedstock, referring to the biomass used, is an essential process parameter in any HTC process. Generally, a higher content of cellulose and hemicelluloses in the biomass leads to an improved oil yield, whereas a higher lignin content results in increased biomass char production. This is due to the complex branching structure of lignin, which makes it more resistant to degradation, thus remaining as a residue.^35^

Research on the thermal utilization of residues from genetically modified sugar cane is notably limited, with even fewer studies focusing on enhancing the energy characteristics of these residues through hydrothermal carbonization.^47−51^ The ability to generalize findings is further constrained due to the genetic modifications tailored to adapt sugar cane to specific soil conditions and production objectives.^52^ In Colombia, sugar cane has undergone extensive genetic alterations, impacting the composition, structure, leaves, and stems of the plant.^53^ Identifying the unique characteristics and behaviors of residues from genetically modified sugar cane in processes such as hydrothermal carbonization is crucial for assessing their potential in energy generation applications. This study introduces a novel approach by examining the hydrothermal carbonization of these residues, providing new insights into the potential for energy production.

In this study, hydrochar was produced from GHR derived from sugar cane through hydrothermal carbonization processes. Due to the limited information on the hydrothermal carbonization of GHR derived from sugar cane, this study aims to evaluate the impact of temperature and H_2_O/GHR ratio on mass and energy yield leading to recycling biomass in an innovative way, so it is expected that this research will provide a beneficial solution to the environmental problems associated with agricultural waste from sugar cane cutting. The hydrochar obtained was characterized by various analytical, spectroscopic, and thermal analysis methods.

## 2 Materials and Methods

Samples of GHRs were collected from the Valle del Cauca department in Colombia. These samples were crushed and screened to achieve a uniform average grain size of 0.356 mm in diameter for further physicochemical characterization. The biomass underwent the HTC process to produce hydrochar, which was then evaluated for its fuel and combustion properties, as well as the behavior of the ash content in the hydrochar derived from the hydrothermal carbonization of GHR.

Different analyses have been conducted to assess the composition of the GHR, focusing on its potential as a substitute for traditional biomasses. Table 1 presents the comparative analysis of GHR versus other sugar-related biomasses, such as bagasse and pith, highlighting their proximate analysis and calorific values. This analysis demonstrates that the general composition of GHR is very close to that of bagasse, the industrial residue left after the extraction of juice from sugar cane, and pith, a derivative of bagasse. This similarity suggests that GHR could be a viable substitute for bagasse currently used in cofiring with coal, without causing significant changes in the characteristics of the raw material.^52^

**Table 1:** Proximate Analysis and Calorific Value of GHR, Bagasse, and Pith, %w/w (db, Dry Basis)

material	GHR	bagasse	pith	ref
residual moisture (%)	6.8	7.5	8.2	(52)
volatile matter (%)	73.7	78.7	86.1	
fixed carbon (%)	9.2	6.3	4.3	
ash (%)	17.1	15.0	9.6	
HHV (kJ/kg)	16,555	16,727	16,425	

Table 2 details the properties of the constituents of GHR, including buds, green leaves, and dry leaves, providing insights into their individual compositions.^54^ While there are similarities in the constituents of GHR, differences are primarily noted in their ash content, which is significant from both energetic and technical perspectives. This variability can affect the overall composition of the GHR depending on local cultivation and harvesting characteristics. These variations are influenced by factors such as the method of collection (mechanical or manual), the type of machinery used (whether it includes bud removal or not), the interval between sugar cane harvest and residue collection, and even the weather conditions during collection.^52^ Understanding these differences is vital for tailoring the HTC process to optimize the yield and quality of hydrochar, ensuring efficient and sustainable biomass utilization.

**Table 2:** Properties of GHR Constituents, %w/w (db)

material	buds	green leaves	dry leaves	ref
residual moisture (%)	6.04	6.22	5.94	(54)
volatile matter (%)	75.79	71.32	72.90	
fixed carbon (%)	16.62	18.29	14.78	
ash (%)	7.59	10.39	12.32	
HHV (kJ/kg)	17,922	16,946	16,825	

### Hydrochar Preparation by HTC

HTC was conducted using a Parr 4848 series autoclave reactor with a 4838 controller at 500 mL capacity, equipped with stirring. The experimental design followed a composite factorial central experimental design as shown in Table 3. In a typical run, 15 g of GHR sample mixed with deionized water was added to the autoclave reactor at water/GHR ratios of 5/1 and 10/1 (w/w). The reactor was then placed in an electric oven with a digital temperature controller. During the pretreatment process, the reactor was maintained at the desired temperature of 200 and 300 °C for 30 min at autogenous pressure, with an agitation speed of 300 rpm. After the predetermined time, the reactor was cooled to room temperature using a cooling coil passing through the reaction zone. The solid sample was washed, collected through vacuum filtration, and dried at 105 °C for 24 h until a constant weight was achieved. The GHR samples were labeled as *H*–*X*–*Y*, where *H* represents the hydrothermal, *X* is the temperature, and *Y* is the value of the water/GHR ratio, according to a central composite factorial experimental design with triple repetition of the central point. The variables in the experimental design were the reaction temperature and the water/GHR ratio.

**Table 3:** Experimental Matrix Design

sample	run	temperature	ratio H_2_O/GHR	coding
1	9	180	7.5/1	H-180–7.5
2	1	200	5/1	H-200–5
3	10	200	10/1	H-200–10
4	7	250	4/1	H-250–4
5	5	250	7.5/1	H-250–7.5(1)
6	2	250	7.5/1	H-250–7.5(2)
7	6	250	7.5/1	H-250–7.5(3)
8	3	250	11/1	H-250–11
9	11	300	5/1	H-300–5
10	4	300	10/1	H-300–10
11	8	321	7.5/1	H-321–7.5

### 2.2 Characterization of GHR and Its Hydrochar

The proximate analysis characteristics, including total moisture, volatile matter, ash, fixed carbon, and gross calorific value (HHV), were determined experimentally by following the respective standard methods: ASTM D3302/D3302M-19 for total moisture, ASTM D7582-15 for volatile matter and ash, ASTM D3172-13 for fixed carbon, and ASTM D5865/D5865M-19 for HHV. The ultimate analysis characteristics, such as carbon, hydrogen, oxygen, and sulfur, were analyzed according to ASTM D5373-21 method A for elemental composition and ASTM D4239-18e1 method A for sulfur, with all values expressed on a dry basis. The Channiwala and Parikh correlation, which provides an alternative method for estimating HHV from biomass elemental (C, H, S, O, N) and ash (A) composition, is shown for comparative purposes in eq 1.^55^ However, this study relied on direct experimental measurements of HHV to ensure accuracy and precision.
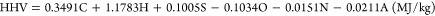
1

The mass yield, energy yield, and energy density of the HTC process can be calculated as follows:

*Mass yield dry basis*
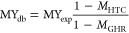
2where MY_db_ = mass yield (dry basis), MY_exp_ = experimental mass yield (the ratio of the mass of hydrochar obtained to the mass of GHR fed into the process, with both weighed on a wet basis), *M*_HTC_ = hydrochar moisture, and *M*_GHR_ = GHR moisture.

*Mass yield dry and ash-free*
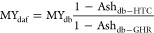
3where Ash_db-HTC_ = hydrochar ash fraction (dry and ash-free) and Ash_db-GHR_ = GHR ash fraction (dry and ash-free)

*HHV fed dry and ash-free*
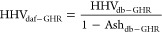
4

*HHV hydrochar was dry and ash-free*
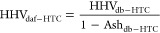
5

*Energy yield*
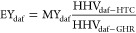
6

*Energy density*
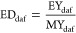
7

### 2.3 Optimization of Hydrochar Production Using Central Composite Design

The research applied a central composite design (CCD) based on the response surface methodology (RSM) to optimize the production of hydrochar as well as its mass and energy yields through the hydrothermal carbonization of GHR. CCD was selected to efficiently create a quadratic polynomial model with a minimal number of experiments. This design allows for a thorough evaluation of interactions between parameters and the identification of the main factors that affect response optimization. CCD is particularly useful in chronological experimental work, as it can build upon earlier factorial experiments through the addition of axial and center points. A CCD can be used to (i) effectively approximate first- and second-order terms and (ii) model a response variable using curvature by adding center and axial points to a factorial design.^56^

RSM is a mathematical and statistical method used to design experiments and optimize the effects of process variables. It models and analyzes situations where multiple variables influence a response of interest. For this study, MATLAB (Mathworks) was employed to conduct the RSM analysis, guiding the use of CCD. Hydrochar was prepared using hydrothermal carbonization, with the input variables of temperature, which ranged from 200 to 300 °C (low and high levels, respectively), and the water/GHR ratio, ranging from 5/1 to 10/1 (low and high levels, respectively), using a design based on the CCD. A 2^2^ compound central factorial design, involving two factors and two levels, was employed for the variables. It included 4 factorial points, 4 axial points, and 3 replicates of the central point, totaling 11 experiments. This design facilitated the development of an empirical model, represented in eq 8. It incorporates an intercept, first-order effects of temperature (*x_i_*) and the H_2_O/GHR ratio (*x_j_*), an interaction term between these effects (*x_i_x_j_*), and their second-order effects (*x_i_*^2^ and *x_j_*^2^, respectively).
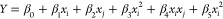
8where *Y* represents the response variable and β is the coefficient of the multiple linear regression fit. β_0_ is the intercept, and β_1_ to β_5_ are regression coefficients calculated from the observed experimental values of Y from experimental runs.

### 2.4 Polymeric Composition Analysis of GHR and Its Hydrochar

**Figure 2:**
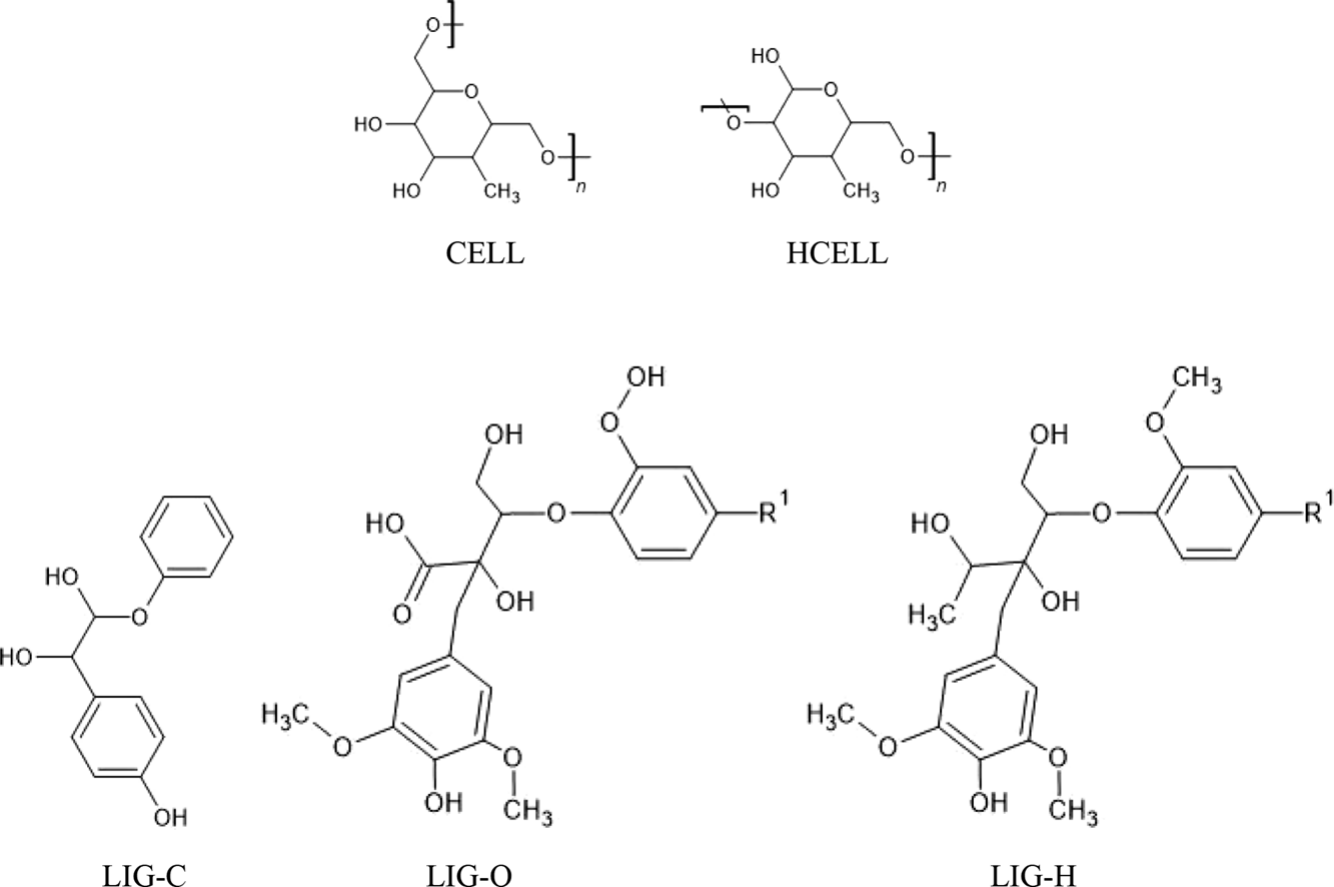
Reference species representing cellulose, hemicellulose, and lignins. (CELL: C_6_H_10_O_5_ (44.44%C-6.17%H-49.38%O), HCELL: C_5_H_8_O_4_ (45.45%C-6.06%H-48.48%O), LIG-C: C_15_H_14_O_4_ (69.77%C-5.43%H-24.80%O), LIG-O: C_20_H_22_O_10_ (56.87%C-5.21%H-37.92%O), and LIG-H: C_22_H_28_O_9_ (60.55%C-6.42%H-33.03%O)).

The biochemical composition of GHR and hydrochars is determined using a mathematical algorithm developed by Debiagi et al.^57^ This methodology identifies the structural composition of the reference species, such as cellulose, hemicellulose, and the three different types of lignins, each illustrated in Figure 2 with their respective elemental compositions of carbon, hydrogen, and oxygen. This method employs elemental analysis as the basis for calculating the content of lignin, cellulose, and hemicellulose. The polymer composition of GHR and hydrochars is then calculated as a linear combination of these theoretical mixtures, providing a detailed approximation of their molecular structure.^52^

## 3 Results and Discussion

### 3.1 Effect of HTC Process Parameters

**Figure 3:**
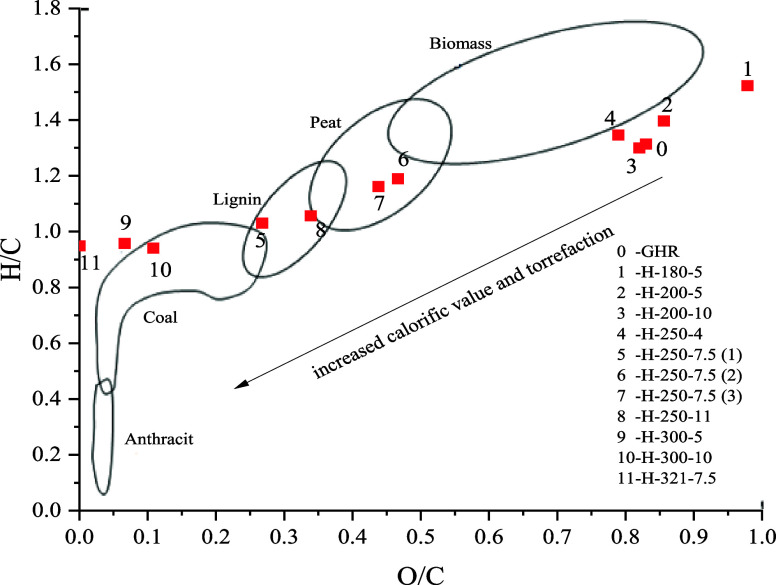
Van Krevelen diagram comparing O/C and H/C atomic ratios of GHR and hydrochars.

The composition results of the proximate, ultimate, and HHV analyses for both the untreated GHR and the hydrochars produced from it are reported on a dry basis and are presented in Table 4. The proximate analysis results indicate that as the HTC temperature increases, volatile matter in the hydrochars decreases, while ash content and fixed carbon increase. Additionally, it is observed that increasing the H2O/GHR ratio at the same temperature leads to a decrease in ash concentration in the hydrochars, due to the leaching effect of water on the minerals present. The ultimate analysis shows that the carbon content in the hydrochars increases with the severity of the HTC process, except in experiments conducted at temperatures of 250 and 300 °C with water/GHR ratios of 7.5/1 and 5/1, respectively. Conversely, the hydrogen and oxygen contents decrease, leading to an improvement in the HHV of the hydrochars. These compositional changes due to hydrothermal treatment are depicted in the Van Krevelen diagram (Figure 3), which illustrates how, under certain treatment conditions, the hydrochars acquire characteristics similar to other carbonaceous materials, such as peat and coal.

**Table 4:** Proximate, Ultimate, and HHV Analyses on a Dry Basis of Untreated GHR and its Hydrochars

samples	proximate analysis (% wt db)				ultimate analysis (% wt db)					HHV_db_ (MJ kg^–1^)
	moisture	volatile matter	ash	fixed carbon	carbon	hydrogen	nitrogen	oxygen	sulfur	
GHR	8.5	59.7	33.2	7.1	34.3	4.4	0.1	27.9	0.22	12.6
H-180–7.5	6.0	64.1	26.7	9.2	39.9	4.6	0.3	28.4	0.08	12.2
H-200–5	5.0	59.8	32.0	8.2	37.5	4.1	0.4	26.0	0.07	15.4
H-200–10	5.9	68.8	22.4	8.8	43.5	4.9	0.6	28.6	0.07	15.3
H-250–4	2.2	34.1	48.0	17.9	39.2	3.4	0.6	8.8	0.07	14.7
H-250–7.5	2.4	35.0	52.0	13.0	36.2	3.3	0.5	8.0	0.08	12.8
H-250–7.5	1.8	24.3	68.4	7.3	20.5	2.0	0.4	8.7	0.06	7.6
H-250–7.5	2.1	24.2	69.4	6.4	23.8	2.3	0.5	4.0	0.08	8.8
H-250–11	2.2	39.0	44.3	16.7	40.2	3.5	0.5	11.4	0.08	15.0
H-300–5	1.2	23.7	62.8	13.5	32.3	2.6	0.4	1.8	0.08	16.2
H-300–10	1.4	29.2	50.2	20.6	42.1	3.3	0.6	3.8	0.09	14.3
H-321–7.5	1.2	28.0	52.4	19.6	47.2	3.7	0.6	0.0	0.08	14.5

Table 5 displays the experimental yields determined by the relationship between the mass of the char obtained after the HTC process and the mass of the GHR; the yields on a dry basis and ash-free basis are determined by eqs 2 and 3 , respectively. This table also reports energy efficiency on a dry and ash-free basis as determined by eq 6, the fuel ratio (content of fixed carbon divided by the content of volatile matter, FC/VM), the H/C and O/C atomic ratios, and finally, the energy density as determined by eq 7. The analysis of the data in Table 5 indicates that the input variables of the process (temperature and H2O/GHR ratio) have significant effects on the experimental yield, mass yield on a dry basis, and mass yield on an ash-free basis. The statistical significance of these results is supported by *F*-values and *P*-values, as detailed in Table 6.

**Table 5:** Mass Yields, Energy Efficiency, Fuel Ratio, H/C and O/C Atomic Ratios, and Energy Density

samples	experimental yield (%)	mass yield db (%)	mass yield daf (%)	energy efficiency daf (%)	flue ratio CF/MV, db	energy density	H/C	O/C
GHR					0.12		1.52	0.61
H-180–7.5	65.3	67.1	71.65	63.03	0.14	0.88	1.40	0.54
H-200–5	64.3	66.7	65.42	78.71	0.14	1.20	1.31	0.52
H-200–10	60.8	62.5	70.54	73.77	0.13	1.05	1.35	0.49
H-250–4	52.3	55.9	40.71	61.17	0.52	1.50	1.03	0.17
H-250–7.5	65.5	69.8	47.05	66.34	0.37	1.41	1.08	0.16
H-250–7.5	62.4	67.0	29.49	37.57	0.30	1.27	1.19	0.32
H-250–7.5	54.9	58.7	25.10	38.44	0.26	1.53	1.16	0.13
H-250–11	49.9	53.3	41.56	59.35	0.43	1.43	1.06	0.21
H-300–5	54.2	58.5	30.16	69.59	0.57	2.31	0.96	0.04
H-300–10	45.1	48.6	33.63	51.21	0.70	1.52	0.94	0.07
H-321–7.5	46.7	50.4	33.28	53.79	0.70	1.62	0.95	–0.06

**Table 6:** Statistical Tests for *F*-values and *P*-values

	*F*-value	*P*-value	
		temperature	ratio H_2_O/GHR
experimental yield	7.59	0.0058	0.283
mass yield db	5.17	0.017	0.275
mass yield daf	8.64	0.0032	0.755

This analysis highlights that while temperature is a statistically significant factor influencing the outcomes of the process (*P*-value <0.05), the H_2_O/GHR ratio does not show a significant impact (*P*-value >0.05). These findings are crucial for optimizing the HTC process, emphasizing temperature control as critical for achieving desired yields and properties in the produced hydrochars.

**Figure 4:**
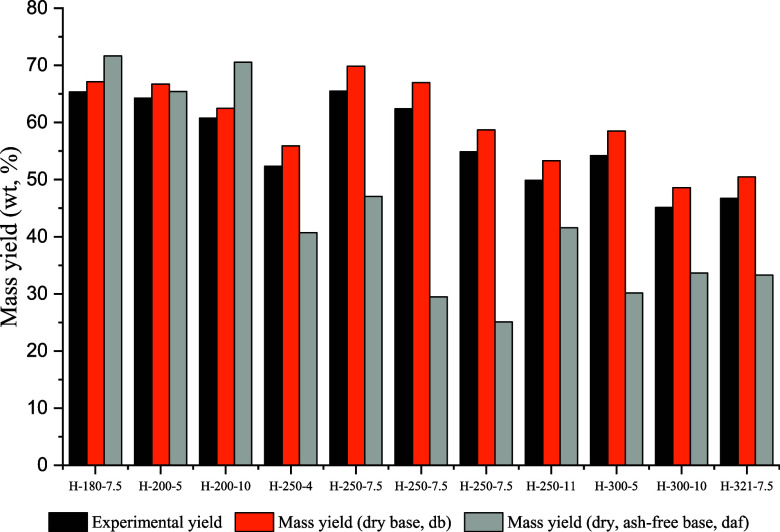
Hydrochar yields as a function of temperature and water/GHR ratios.

**Figure 5:**
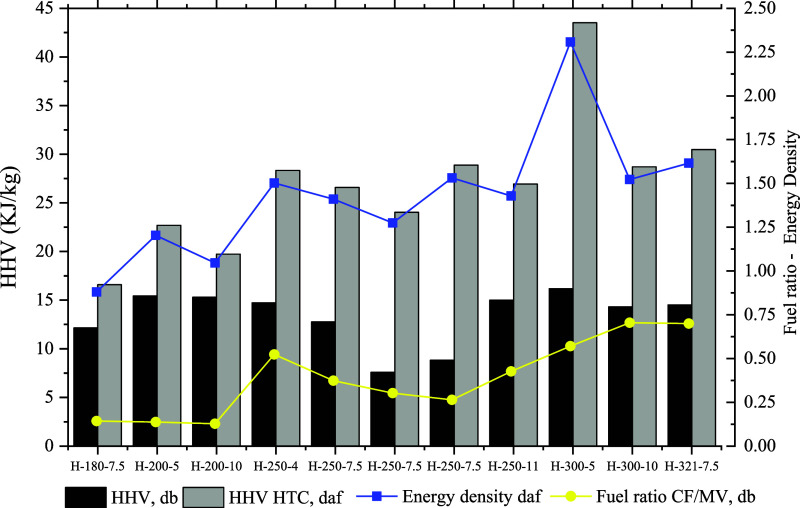
HHV, fuel ratio, and energy density of GHR and hydrochars treated.

The results of the mass yields of hydrochar for different temperatures during the HTC process are shown in Figure 4, and the energy parameters are presented in Figure 5. The mass yields of HTC hydrochars, as depicted in Figure 4, are influenced by both the feedstock type and the processing temperatures. The literature on HTC of lignocellulosic biomass reports typical yields ranging between 60 and 67% at lower process temperatures (≤200 °C), between 50 and 70% at a medium process temperature of 250 °C, and between 45 and 58% at higher process temperatures (≥300 °C). In this study, it was observed that yields at lower experimental temperatures of 180 and 200 °C were higher than those obtained at temperatures equal to or exceeding 300 °C. This higher yield at lower temperatures supports the notion that there is limited transformation of biomass into hydrochar at temperatures around 200 °C, aligning with findings by Debiagi et al.^58^ The reduction in yield observed between 200 and 300 °C can be attributed to increased degradation of the organic material and removal of inorganic content.^59^

The energy yield (EY) of the HTC hydrochars, as shown in Figure 5, is influenced by two main factors: mass yield and energy densification, which is the relative increase in the calorific value. As the HTC temperature increases, the mass yield decreases, while energy densification increases, as detailed in Table 5. The EY values across the entire temperature range vary between 37.57 and 78.71%, with the highest value observed for hydrochar H-200–5. Notably, hydrochar H-300–5 displays the highest energy density, achieving a value of 2.31, which correlates with its high HHVdaf, as supported by various studies.^36,40^ In this figure, it is also evident that HHVdb is linked to the CF/MV fuel ratio, and HHVdaf correlates with the energy density.

The HHV (higher heating value of hydrochar) increases as the carbon content increases and the oxygen content decreases. This trend is attributed to the higher energy density of carbon-rich compounds than those rich in oxygen. Carbon-rich materials have a higher proportion of carbon–carbon and carbon–hydrogen bonds, which release more energy upon combustion than the carbon–oxygen bonds prevalent in oxygen-rich materials.^52^ Moreover, the relationship between the proximate analysis composition and HHV indicates that materials with higher fixed carbon (FC) content exhibit increased HHV due to the high energy potential of fixed carbon, which predominantly consists of carbon available for combustion. Conversely, an increase in volatile matter (VM) typically decreases in HHV because these compounds have a lower energy content. These findings are supported by Parnthong et al.^41^ Furthermore, it is observed that a lower ash content correlates with higher HHV. Ash represents incombustible material within the biomass; thus, a higher ash content dilutes the combustible portion, reducing the calorific value.

An experimental expression for the HHV on a dry basis has been derived by correlating volatile matter and fixed carbon from the proximate analysis along with the O/C and H/C ratios from the ultimate analysis. This correlation has resulted in a fit with a coefficient of determination (R2) of 0.94 for experimental data at temperatures ranging from 180 to 320 °C. Additionally, this correlation was compared with that obtained using eq 1 by Channiwala and Parikh,^55^ which also calculates HHV. The comparison shows that eq 1 aligns well with the experimental results of this study, yielding a correlation fit of R2 = 0.95 for temperatures between 200 and 300 °C.
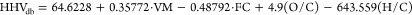
9

Furthermore, the moisture content (*M*) of hydrochar decreases with increasing the temperature. This reduction is primarily due to the dehydration reactions occurring during the carbonization process, which remove water bound within the biomass structure. An expression (eq 10) specifically for calculating the moisture content of hydrochar has been derived using the ash content, H/C ratio, and O/C ratio. Developed from experimental data, this expression is consistent with other formulations in the study, such as eq 9 for HHV, and yields a correlation coefficient (*R*^2^) of 0.96.
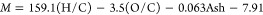
10

The polymeric composition analysis in Table 7 reveals a high lignin content in biomass, which contributes to the formation of solid fuels. Additionally, as the temperature increases, the lignin content increases while the cellulose and hemicellulose contents decrease due to the reactions occurring during HTC.^39^ This includes the solubility that hemicellulose undergoes at lower temperatures, notably between 170 and 180 °C.^60,61^

**Table 7:** Composition of Polymeric Compounds in GHR and Hydrochar at 180 and 200 °C

	celullose	hemicelullose	lignin-O	lignin-C	lignin-H
GHR	0.36	0.24	0.11	0.08	0.21
H-180–7.5	0.25	0.16	0.22	0.12	0.25
H-200–5	0.19	0.13	0.32	0.13	0.22
H-200–10	0.17	0.12	0.53	0.14	0.04

### 3.2 Optimization of HTC Temperature and Water/GHR Ratio

CCD was employed to establish correlations between the temperature and water/GHR ratio to determine the most favorable conditions for hydrochar production, as illustrated in Figures 4 and 5. The response variables examined included maximizing experimental mass yield, mass yield on a dry basis, and HHV, along with minimizing ash content. The coefficients for multiple linear regression related to these variables are presented in Table 8, while the most favorable settings derived from our models are given in Table 9.

**Table 8:** Constants for Multiple Linear Regressions

parameter	β_0_	β_1_	β_2_	β_3_ × 10^3^	β_4_ × 10^3^	β_5_ × 10^1^	*R*^*2*^
ash	–485.6	3.37	28.6	–6.12	–9.58	–18.44	0.96
mass yield	–11.41	0.50	7.03	–1.48	15.0	–7.043	0.92
mass yield, db	–84.05	0.82	17.7	–1.67	–13.5	–10.17	0.94
HHV, MJ/kg	105.2	–0.54	–7.8	1.16	–4.09	5.836	0.94

**Table 9:** Optimal Process Variables

parameter	*T*_opt_ (°C)	*R*_opt_ (water/GHR ratio)
ash	270.0	7.0/1
mass yield	207.1	7.2/1
mass yield, db	217.0	7.2/1
HHV (MJ/kg)	247.2	7.5/1

To effectively define the most favorable HTC process conditions for sugar cane GHR, two primary objectives were outlined: first, maximizing the HHV value and the mass yields on experimental and dry bases; second, achieving the lowest possible ash content in the hydrochar matrix. These objectives aim to enhance the energy efficiency and maximize the useful output of the hydrochar.

**Figure 6:**
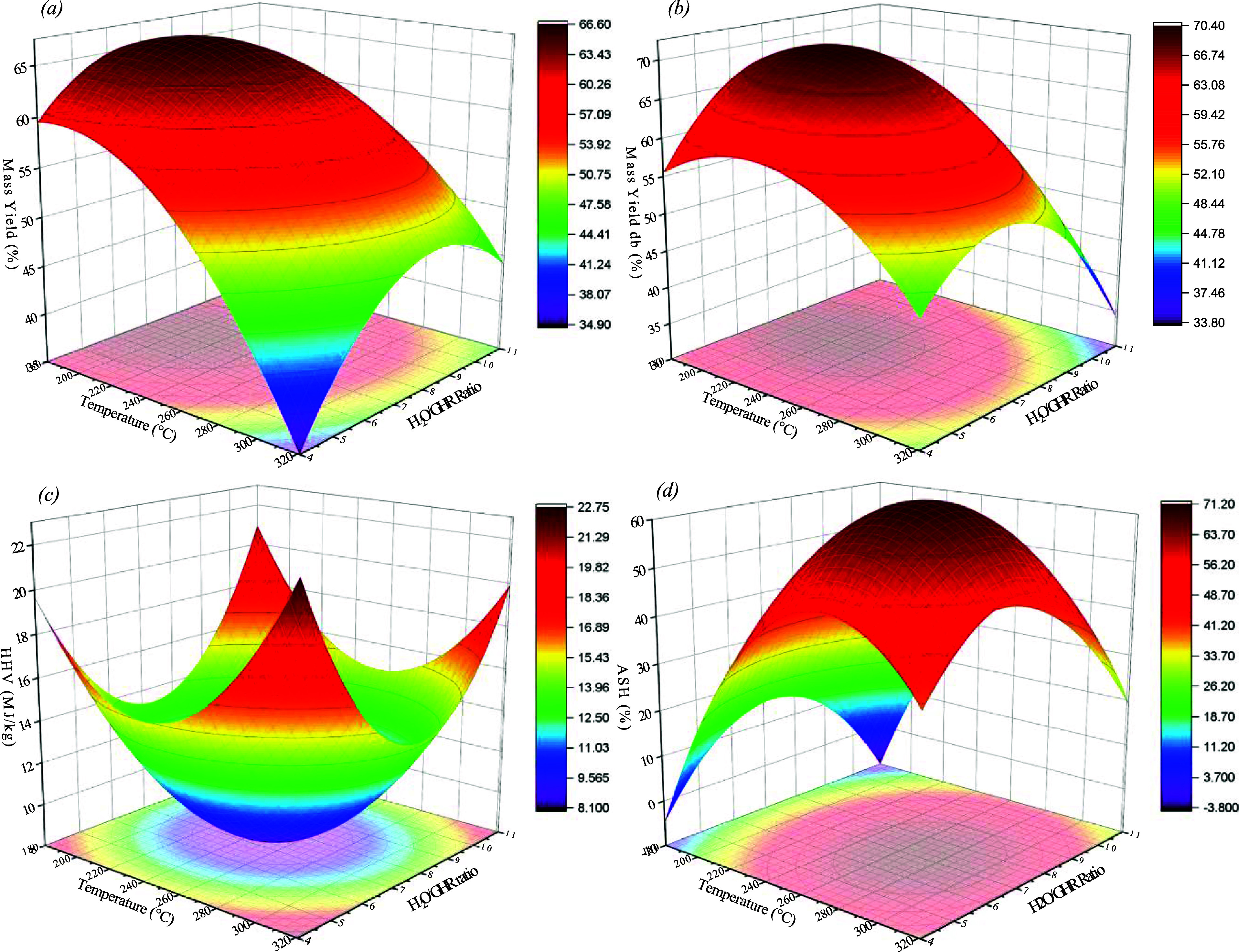
Three-dimensional response surface (effect of temperature and water/GHR ratio) on (a) mass yield; (b) mass yield, db; (c) HHV; and (d) ash.

Figure 6a,b depicts the response surfaces for experimental mass yield and mass yield on a dry basis, indicating values at 207.1 °C for temperature and a water/GHR ratio of 7.2/1. At these lower temperatures, the transformation of GHR into hydrochar involves less decomposition of volatile compounds, leading to a higher retention of the original solid matrix. This retention is indicative of a milder transformation process that conserves more of the inherent biomass structure and energy content. Figure 6c represents the response surface for HHV on a dry basis, showing a higher energy value at a temperature of 247.2 °C and a water/GHR ratio of 7.5/1. Given these findings, it is advisable to extend the experimental design toward the maximum HHV value observed at 300 °C with the same water/GHR ratio. Consideration should also be given to a temperature variation of ±20 °C to effectively reassess its thermal properties.

Similarly, Figure 6d analyzes the behavior of the ash content and reveals a maximum value at 270 °C with a water/GHR ratio of 7/1. Although this condition shows a peak, the objective within the HTC process is to minimize ash content in order to increase the fixed carbon content, thereby enhancing the HHV value. Therefore, conditions at the extremes of the operational ranges evaluated should be explored. This approach aims to find conditions that ensure lower ash content, which is favorable for optimizing HHV.

## 4 Conclusions

The study has demonstrated that agricultural residues from sugar cane, termed GHRs, can effectively be converted into hydrochar via HTC. This transformation is significantly influenced by the temperature and water/GHR ratio, which directly affect the yield and physicochemical properties of the hydrochar. Temperature has been identified as the most critical factor influencing hydrochar quality, particularly impacting carbon content and atomic ratios of O/C and H/C. Consequently, the HHV of hydrochar increased from 18.9 to 43.5 MJ/kg, marking a substantial improvement compared with the original GHR. This increase reflects significant energy densification facilitated by carbon enrichment and reduced oxygen content during HTC.

Response surface analysis was employed to investigate the effects of the temperature and water/GHR ratio on hydrochar properties. This analysis suggested that higher temperatures potentially enhance HHV and reduce ash content, with the most favorable results shifting toward the higher end of the temperature spectrum examined. Given these findings, it is recommended to extend the range of temperature conditions in future studies to further explore and possibly maximize HHV while minimizing the ash content.

Furthermore, while the HTC process alters the physicochemical structure of the GHR, resulting in a product with enhanced properties, the hydrochar notably preserves the intrinsic carbon framework of the original biomass. This retention and enhancement of carbon content contribute to the hydrochar’s increased calorific value, making it a promising material for energy production. These findings underline the effectiveness of HTC in enhancing the energy properties of sugar cane residues, suggesting that hydrochar can potentially replace or supplement conventional fuels in various applications. The results also provide a foundation for further research on expanding the conditions under which HTC is performed to maximize energy yield and minimize undesirable by-products such as ash.
